# Supplementary material for: Intracytoplasmic membranes develop in Geobacter sulfurreducens under thermodynamically limiting conditions
Source: NPJ Biofilms Microbiomes. 2023 Apr 7;9:18. doi: 10.1038/s41522-023-00384-6 (PMC10082016; doi:10.1038/s41522-023-00384-6)
Supplement: Supplementary file 1 — Supplemental Material [file 41522_2023_384_MOESM1_ESM.pdf]

Supplemental Information

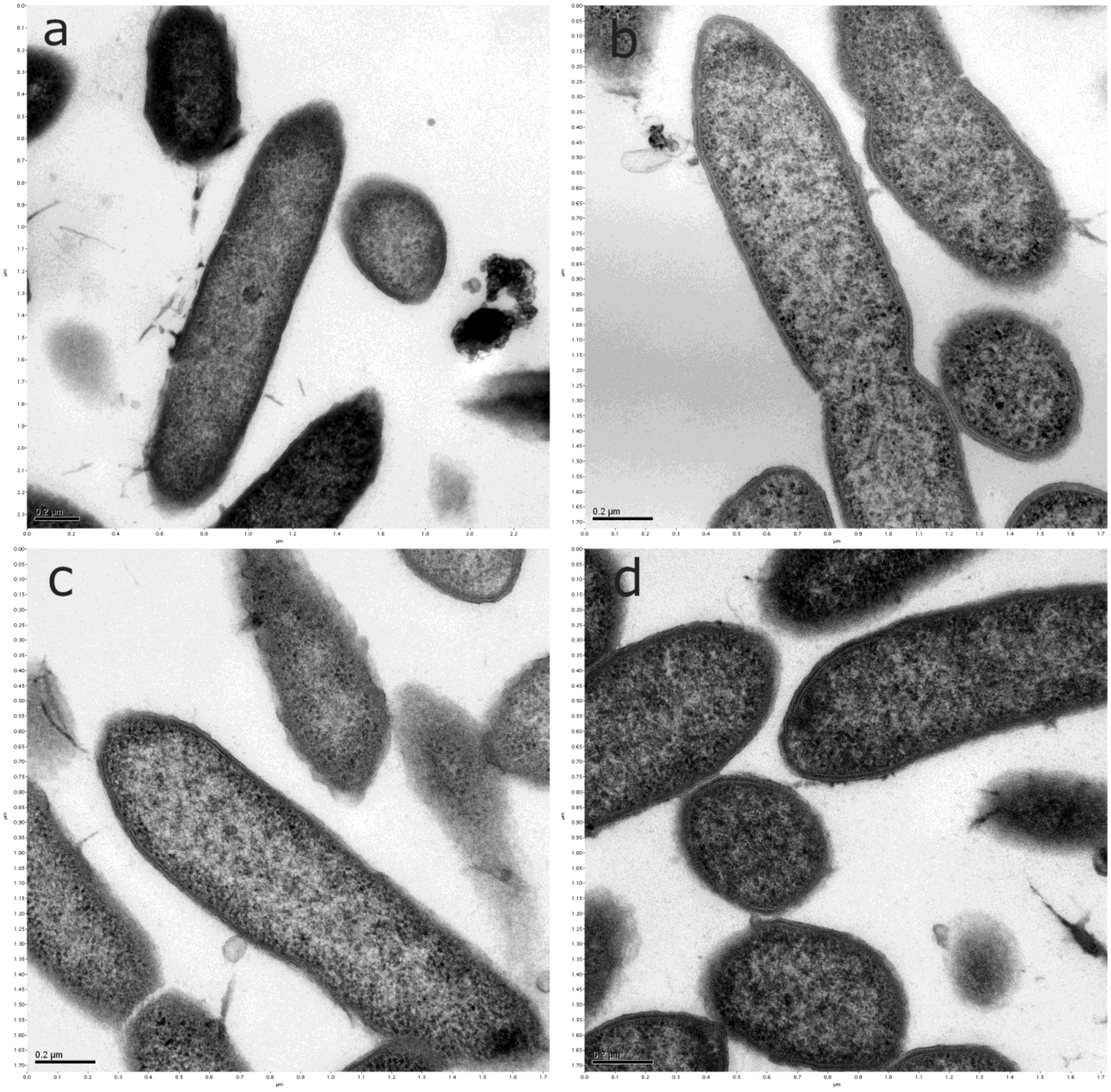

Supplemental Figure 1: (a-d) Conventional thin section TEM micrograph of *G. sulfurreducens* cells grown using fumarate as the electron acceptor. No ICM was observed.

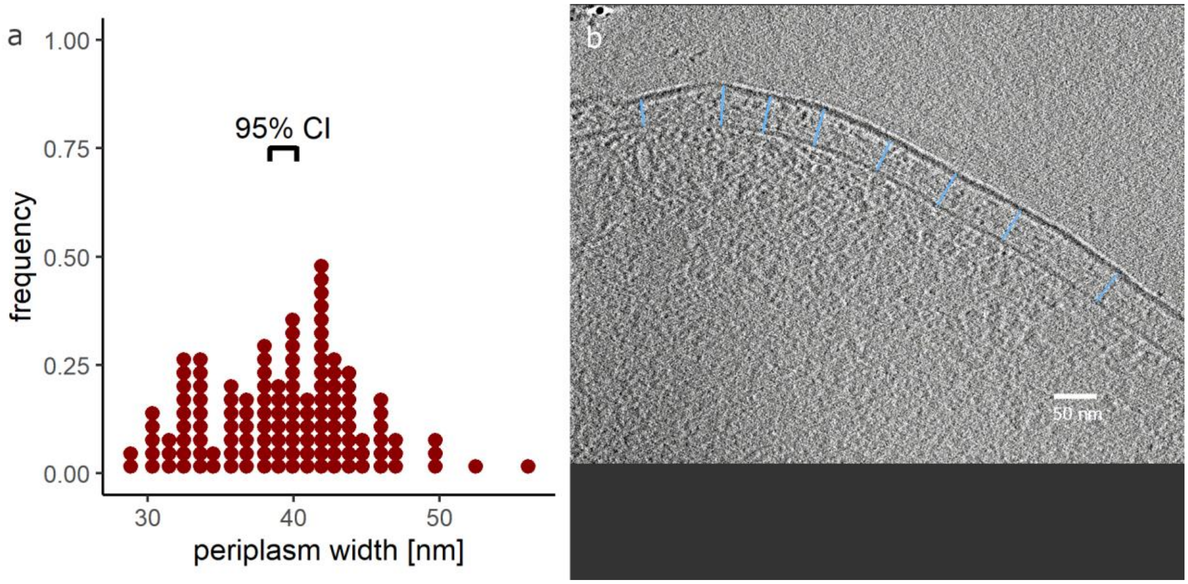

<u>Supplemental Figure 2: </u>Periplasm measurements in *G. sulfurreducens* cryotomograms. (a) dot plot showing distribution of periplasm measurements (n=128); (b) cryotomogram demonstrating how we collected periplasmic measurements by annotating lines from the inner to outer membrane at multiple points on multiple cells.

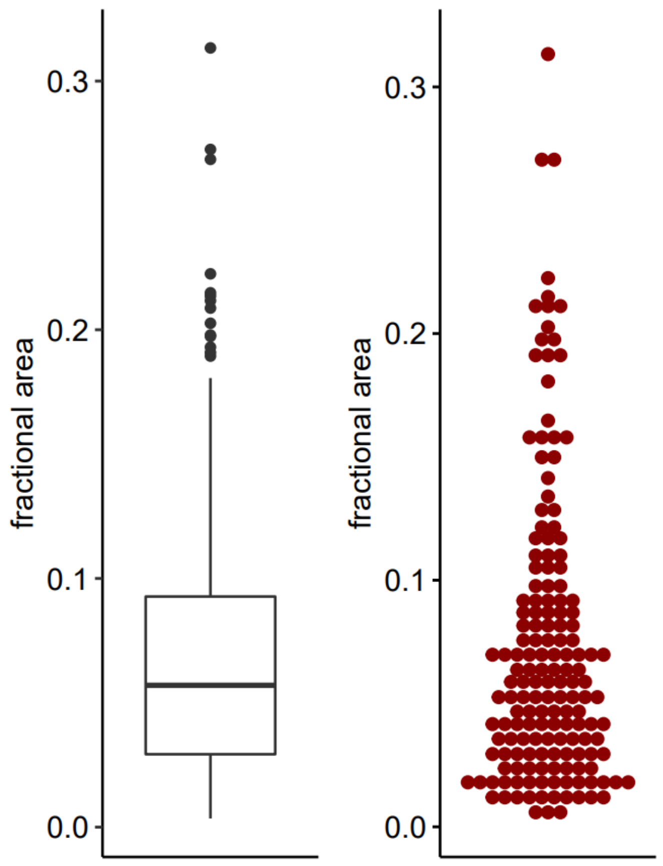

Supplemental Figure 3: Fractional area of cell area occupied by ICM in sum projections of confocal z-stacks collected using Nile red, a lipid stain. Each dot represents a cell (n=164). Every cell represented here came from an electrode biofilm poised at -0.07 V vs. SHE. ICM area and cell area were calculated by MicrobeJ as described in the methods. (Left) box plot, (right) dot plot. Box plot quartile breaks are at 0.029, 0.057, and 0.093.

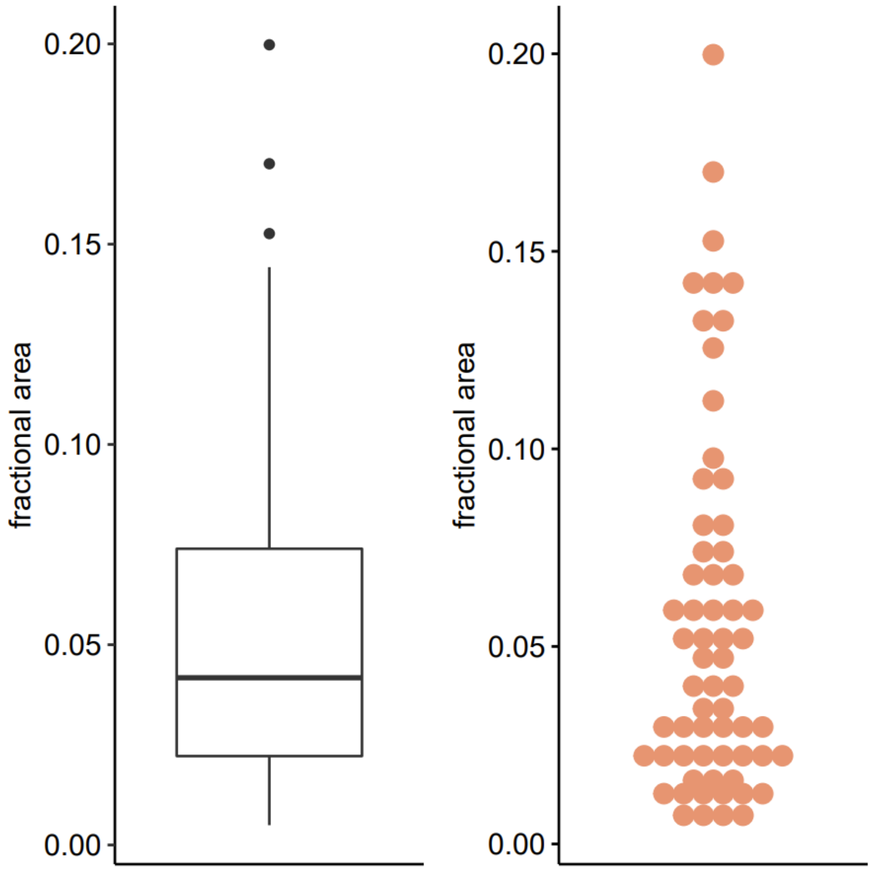

Supplemental Figure 4: Fractional area of cell area occupied by ICM in sum projections of confocal z-stacks collected using Nile red, a lipid stain. Each dot represents a cell (n=63). Every cell represented here came from planktonic cultures grown with 50 mM fumarate as the electron acceptor. ICM area and cell area were calculated by MicrobeJ as described in the methods. (Left) box plot, (right) dot plot. Box plot quartile breaks are at 0.022, 0.042, and 0.074.

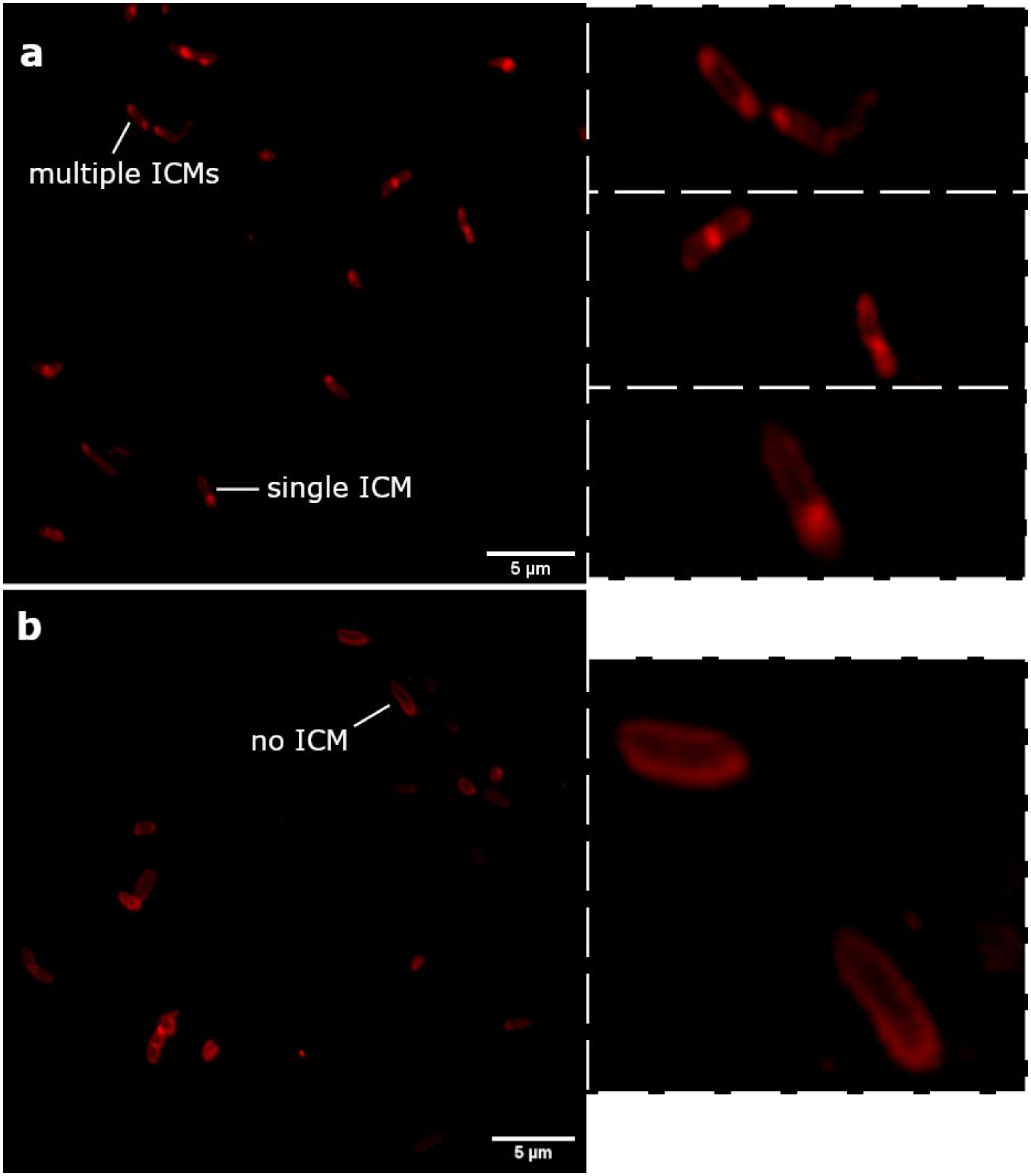

Supplemental Figure 5: Inset of Figure 4 from the main text with a high zoom applied to selected individual cells displaying multiple, one, or no ICM. See Figure 4 for full caption.

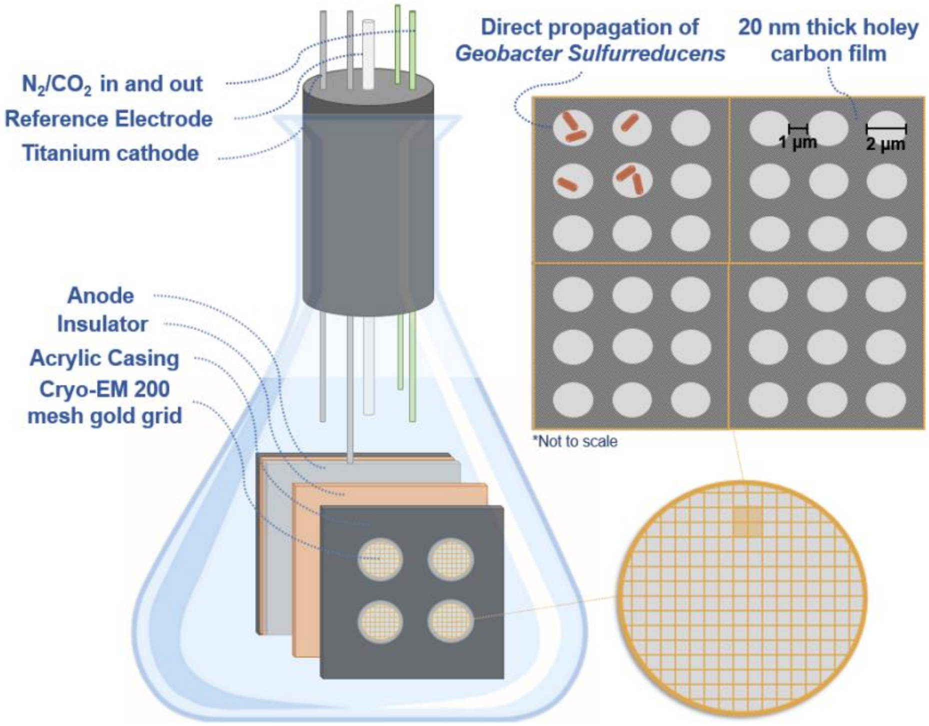

Supplemental Figure 6: Cartoon depicting the design elements of the bioreactor grid holder that is poised as an anode to capture cells in an active state for cryoET. The cartoon is not to scale, and a picture of the actual holder is in Figure S8

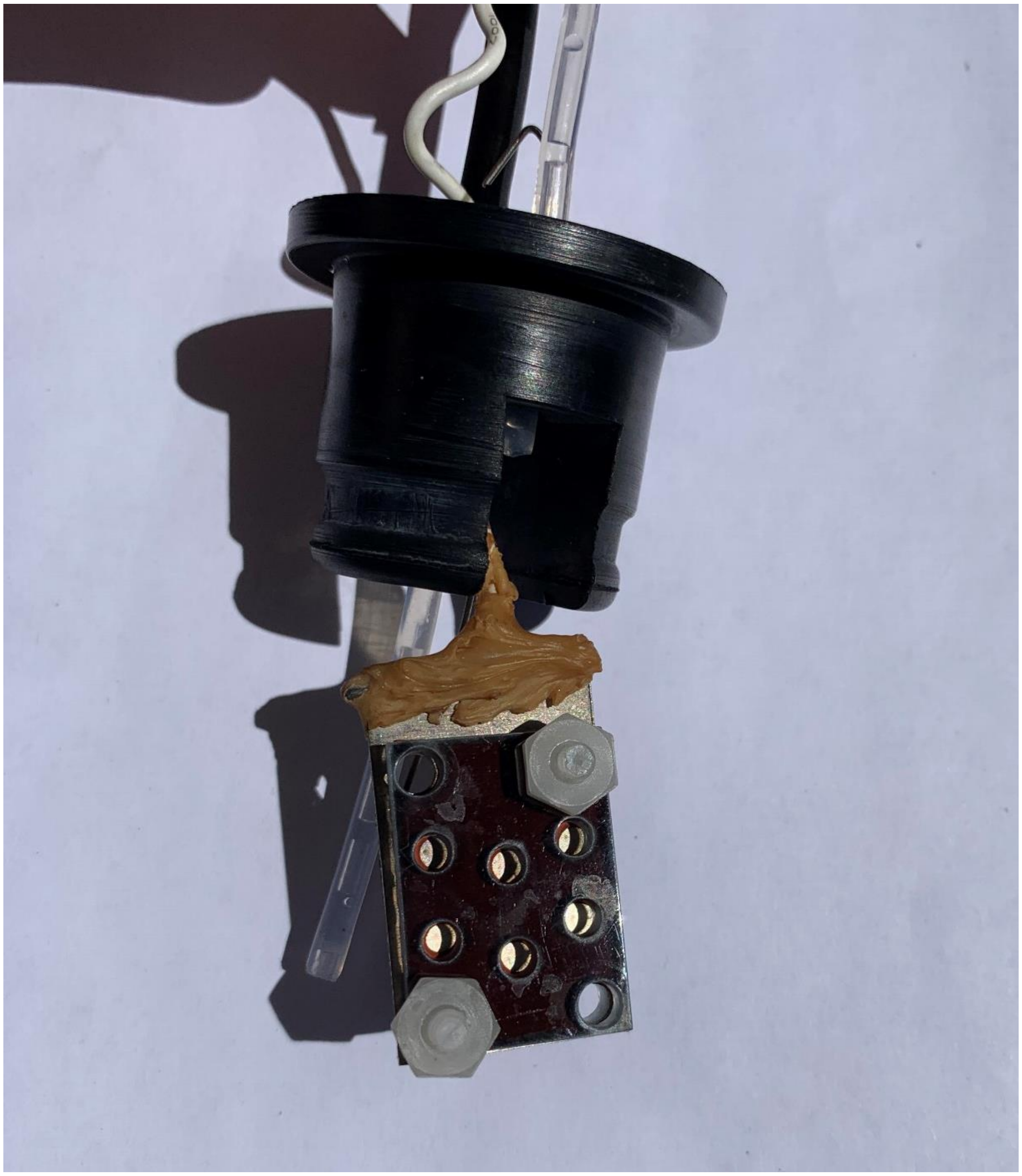

Supplemental Figure 7: Photograph of the grid holder used in a microbial electrochemical cell to grow *G. sulfurreducens* directly on EM grids for cryoET preparation.

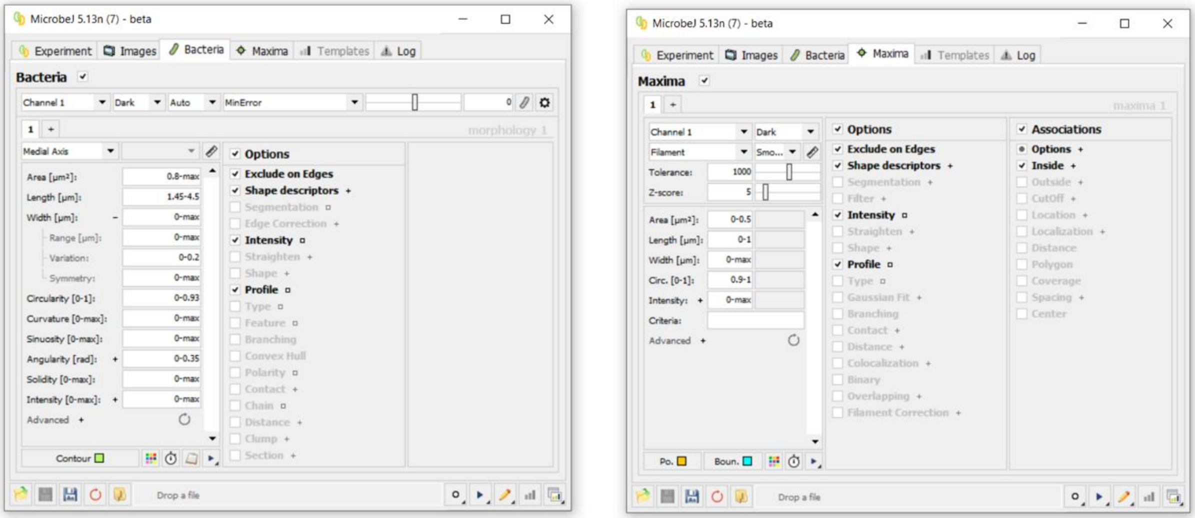

<u>Supplemental Figure 8:</u> Parameters used in the MicrobeJ plugin for ImageJ to detect and quantify ICM in *G. sulfurreducens* cells imaged at 100X with confocal microscopy.

Supplemental Table 1: Summary of the number of cells detected and the fraction of those cells that had detectable ICM in all the images used to create Figure 4a. Cell and local maxima detection were performed with MicrobeJ in ImageJ.

Image Name	No. cells detected	type	No. of cells with ICM	Fraction with ICM
1-3-004	46	Electrode -0.07V	32	0.696
1-3-006	52	Electrode -0.07V	22	0.423
1-3-010	77	Electrode -0.07V	50	0.649
1-12-001	223	Electrode -0.07V	87	0.390
1-12-002	217	Electrode -0.07V	88	0.406
3-30-001	86	Electrode -0.07V	51	0.593
3-30-002	70	Electrode -0.07V	36	0.514
3-30-003	68	Electrode -0.07V	45	0.662
3-30-004	69	Electrode -0.07V	44	0.638
3-30-005	81	Electrode -0.07V	41	0.506
3-30-006	82	Electrode -0.07V	37	0.451
3-30-007	90	Electrode -0.07V	53	0.589
3-30-008	100	Electrode -0.07V	54	0.540
3-30-009	93	Electrode -0.07V	60	0.645
3-30-010	66	Electrode -0.07V	34	0.515
3-30-011	66	Electrode -0.07V	37	0.561
1-21-008a	67	Fumarate 50mM	24	0.358
1-21-009	37	Fumarate 50mM	1	0.027
1-24-011	56	Fumarate 50mM	10	0.179
1-24-014	227	Fumarate 50mM	37	0.163
1-26-001	188	Fumarate 50mM	29	0.154
1-26-002	63	Fumarate 50mM	8	0.127
1-26-005	47	Fumarate 50mM	5	0.106
3-4-006	67	Fumarate 50mM	0	0.000
3-4-001	14	Fumarate 50mM	2	0.143
3-18-006	119	Electrode -0.17V	56	0.504
3-30-001	75	Electrode -0.17V	36	0.560
3-30-002	69	Electrode -0.17V	43	0.710
3-30-003	88	Electrode -0.17V	39	0.545
3-30-004	102	Electrode -0.17V	51	0.569
5-9-003	234	Electrode -0.03V	107	0.457
5-9-004	182	Electrode -0.03V	75	0.412
5-9-006	122	Electrode -0.03V	58	0.475
5-9-007	168	Electrode -0.03V	70	0.417
5-9-008	282	Electrode -0.03V	99	0.351

5-9-009	259	Electrode -0.03V	98	0.378
5-9-010	141	Electrode -0.03V	56	0.397
5-9-011	215	Electrode -0.03V	80	0.372
