# Supplementary material for: Intracytoplasmic membranes develop in Geobacter sulfurreducens under thermodynamically limiting conditions
Source: NPJ Biofilms Microbiomes. 2023 Apr 7;9:18. doi: 10.1038/s41522-023-00384-6 (PMC10082016; doi:10.1038/s41522-023-00384-6)
Supplement: Supplementary file 2 — Reporting Summary [file 41522_2023_384_MOESM2_ESM.pdf]

nature portfolio

Corresponding author(s): Cesar Torres

Last updated by author(s): Jan 17, 2023

Reporting Summary

Nature Portfolio wishes to improve the reproducibility of the work that we publish. This form provides structure for consistency and transparency in reporting. For further information on Nature Portfolio policies, see our Editorial Policies and the Editorial Policy Checklist.

Statistics

For all statistical analyses, confirm that the following items are present in the figure legend, table legend, main text, or Methods section.

n/a	Confirmed	
☐	☒	The exact sample size (*n*) for each experimental group/condition, given as a discrete number and unit of measurement
☐	☒	A statement on whether measurements were taken from distinct samples or whether the same sample was measured repeatedly
☐	☒	The statistical test(s) used AND whether they are one- or two-sided<br>*Only common tests should be described solely by name; describe more complex techniques in the Methods section.*
☐	☒	A description of all covariates tested
☐	☒	A description of any assumptions or corrections, such as tests of normality and adjustment for multiple comparisons
☐	☒	A full description of the statistical parameters including central tendency (e.g. means) or other basic estimates (e.g. regression coefficient) AND variation (e.g. standard deviation) or associated estimates of uncertainty (e.g. confidence intervals)
☐	☒	For null hypothesis testing, the test statistic (e.g. *F*, *t*, *r*) with confidence intervals, effect sizes, degrees of freedom and *P* value noted<br>*Give P values as exact values whenever suitable.*
☒	☐	For Bayesian analysis, information on the choice of priors and Markov chain Monte Carlo settings
☒	☐	For hierarchical and complex designs, identification of the appropriate level for tests and full reporting of outcomes
☒	☐	Estimates of effect sizes (e.g. Cohen's *d*, Pearson's *r*), indicating how they were calculated

Our web collection on statistics for biologists contains articles on many of the points above.

Software and code

Policy information about availability of computer code

Data collection	Images were collected at a nominal magnification of 6500x giving a 1.8 Å pixel size in super-resolution mode on the K2 summit camera with a dose rate of 0.5 electron per Å2 * second for three seconds with a frame rate of 0.2 frames per second (total dose of 100 electrons per Å2). Individual movie frames were gain corrected, aligned with MotionCor255, and sum images were binned by 2 resulting in an image with a 3.6 Å/pixel scale. Summed images were restacked, then tomographic reconstruction in eTOMO56 was performed. Slices from each tomogram were output from IMOD using the ZAP window saving feature which saves at the native resolution of the monitor. 3D modeling was performed in IMOD and visualized in ChimeraX57.<br>Confocal data collection was performed with Nikon Elements software.
Data analysis	R Version 4.1.3 was used for statistical analysis and plotting.<br>IMOD was used to create 3D models from tomograms.<br>Fluorescence image analysis was performed in ImageJ with the MicrobeJ plugin.

For manuscripts utilizing custom algorithms or software that are central to the research but not yet described in published literature, software must be made available to editors and reviewers. We strongly encourage code deposition in a community repository (e.g. GitHub). See the Nature Portfolio guidelines for submitting code & software for further information.

Data

Policy information about availability of data

All manuscripts must include a data availability statement. This statement should provide the following information, where applicable:

- Accession codes, unique identifiers, or web links for publicly available datasets
- A description of any restrictions on data availability
- For clinical datasets or third party data, please ensure that the statement adheres to our policy

The tomograms used to create figures for this manuscript can be accessed in the EMDB-EBI repository under accession numbers EMD-27710, EMD-27729, EMD-27748, and EMD-27747.

Human research participants

Policy information about studies involving human research participants and Sex and Gender in Research.

Reporting on sex and gender	NA
Population characteristics	NA
Recruitment	NA
Ethics oversight	NA

Note that full information on the approval of the study protocol must also be provided in the manuscript.

Field-specific reporting

Please select the one below that is the best fit for your research. If you are not sure, read the appropriate sections before making your selection.

☒ Life sciences ☐ Behavioural & social sciences ☐ Ecological, evolutionary & environmental sciences

For a reference copy of the document with all sections, see nature.com/documents/nr-reporting-summary-flat.pdf

Life sciences study design

All studies must disclose on these points even when the disclosure is negative.

Sample size	No pre-experiment sample size calculation was performed. For electron microscopy, no statistical analysis was performed. For fluorescence analysis, each image of dozens of cells was treated as a sample to apply the central limit theorem, and a minimum of 5 images was used for each condition.
Data exclusions	MicrobeJ image analysis parameters were set to exclude abnormally shaped cells, and the same parameters were applied to all images.
Replication	Our experimental design used true biological replicates as well as technical replicates, and multiple imaging events.
Randomization	We did not use randomization, as it does not really apply to bacterial culture. We assume that since we used the same strain of organism in all experiments, there should not be significant variance in our population from anything besides our manipulations.
Blinding	No blinding was applied to our study. We did use the same software parameters on each confocal microscopy image to remove potential bias from the lack of blinding.

Reporting for specific materials, systems and methods

We require information from authors about some types of materials, experimental systems and methods used in many studies. Here, indicate whether each material, system or method listed is relevant to your study. If you are not sure if a list item applies to your research, read the appropriate section before selecting a response.

Materials & experimental systems

n/a	Involved in the study
☒	☐ Antibodies
☒	☐ Eukaryotic cell lines
☒	☐ Palaeontology and archaeology
☒	☐ Animals and other organisms
☒	☐ Clinical data
☒	☐ Dual use research of concern

Methods

n/a	Involved in the study
☒	☐ ChIP-seq
☒	☐ Flow cytometry
☒	☐ MRI-based neuroimaging
